# A comprehensive dataset for uncovering housing price drivers in emerging cities: Nanning as a case study

**DOI:** 10.1016/j.dib.2026.112884

**Published:** 2026-05-25

**Authors:** Wenbo Lin, Kaichuang Wu, Xiaolu Zhang, Xiaoling Gu, Ming Jiang, Jun Wang, Feng Han

**Affiliations:** a School of Artificial Intelligence, Guangxi University for Nationalities, No.188 Daxue East Road, Nanning, Guangxi 530006, China; b Guangxi Key Laboratory of Hybrid Computation and IC Design Analysis, No.188 Daxue East Road, Nanning, Guangxi 530006, China; c Guangdong Academy of Environmental Science, No.335 Dongfeng Zhong Road, Yuexiu District, Guangzhou, Guangdong 51000, China; d College of Resources and Environment, Beibu Gulf University, No.12 Binhai Avenue, Qinzhou, Guangxi 535011, China

**Keywords:** Housing prices, Geospatial data, SHAP, Random forest, Urban sustainability, Machine learning

## Abstract

This dataset supports the research article titled "Exploring housing price dynamics in sustainable cities through a cooperated big data driven machine learning method: case study on a typical city in China", published in *City and Environment Interactions*. The data were collected from multiple sources, including web-scraped real estate listings, air quality monitoring stations, public amenities using the Gaode Map API, and population data from the LandScan global dataset. The dataset includes variables describing property characteristics, accessibility, environmental quality, and land use patterns. Random Forest modeling and SHAP values were used to interpret the contribution of each feature to housing price volatility. This dataset is valuable for urban economists, planners, and data scientists studying housing market dynamics, land use policy, or spatial machine learning. It enables replication, benchmarking, and comparative studies in similar urban contexts across developing cities.

Specifications Table

**Table:** 

Category	Description
Subject	Earth and Environmental Sciences
Specific subject	Urban housing market, real estate valuation
Type of data	Table, Figure, GeoJSON, CSV
Data formats	Raw, Cleaned, Processed
How data were acquired	Real estate listings were scraped from major platforms for 2018 (anchor year), spatial facility data were obtained via Gaode Map API, AQI was interpolated from seven government monitoring stations using IDW, and population density was extracted from LandScan 2023 as the most recent standardized gridded population product with full citywide coverage.
Data source location	Nanning, Guangxi, China (22.817° N, 108.366° E)
Data accessibility	Repository: han, feng (2025), “Price and basic information of residential units in Nanning”, Mendeley Data, V4, Doi: 10.17632/pj2zff4p9m.1
Related research article [1]	Han, F., Lu, M., Qin, D., Zheng, G., Zeng, G., Tan, Y., Wu, Z., Lu, H., Wang, J., Deng, Y., & He, H. (2025). Exploring housing price dynamics in sustainable cities through a cooperated big data driven machine learning method: case study on a typical city in China. City and Environment Interactions, 28, 100,223. https://doi.org/10.1016/j.cacint.2025.100223

## 1 Value of the Data

This dataset offers high-resolution spatial housing data, incorporating 2508 residential communities across Nanning with detailed attributes collected through web crawling and GIS-derived indicators. Such granularity allows researchers to conduct fine-scale spatial analyzes of housing price dynamics, which is particularly valuable for urban planning and socioeconomic studies.

By integrating machine learning and explainable AI techniques, the dataset supports robust predictive modeling using Random Forest while enhancing model interpretability through SHAP values. This approach helps uncover nonlinear relationships and provides insights into the marginal contribution of each feature to housing price predictions.

The inclusion of policy-relevant features, such as proximity to public amenities, land-use restrictions, and environmental attributes, enables researchers and planners to examine how governance tools and regulatory interventions influence housing affordability and price volatility. The dataset can also be used to replicate the analytical framework and modeling strategy adopted in the related research article, facilitating robustness checks and methodological validation.

By highlighting areas with price volatility and standard deviation anomalies, the dataset facilitates spatial housing risk assessments. These insights are critical for developing investment strategies, identifying vulnerable urban areas, and informing sustainable development efforts. In addition, the standardized structure of the dataset allows comparative analyzes across different cities or regions when similar variables are available.

The dataset is derived from Nanning, a benchmark case for rapidly urbanizing cities. Beyond research applications, the dataset is suitable for educational use in applied statistics, spatial analysis, and urban economics courses, where it can serve as a practical example for data preprocessing, modeling, and result interpretation.

## 2 Background

Understanding housing price dynamics is crucial for achieving sustainable urban development, particularly in rapidly growing cities such as Nanning, China. Housing markets are shaped by a complex interplay of structural, cyclical, geographical, and institutional factors [2]. Structural aspects—such as building quality, age, and unit size—interact with broader economic trends like income levels and credit availability to shape demand. Meanwhile, spatial accessibility to amenities, public transit, and environmental quality significantly influences perceived value and pricing [3,4].

To capture housing market variations at a fine spatial scale, this dataset uses the "residential community" as the basic unit of analysis (i.e., a named housing estate/neighborhood unit identified by community name and geographic coordinates), and housing price is summarized as the average price within each residential community.

Traditional economic models often fall short in capturing the non-linear interactions among these variables [5]. In contrast, machine learning (ML) approaches, especially ensemble methods like Random Forest (RF), are better suited to handle data heterogeneity and complex feature interactions [6]. When combined with interpretability techniques such as SHAP (SHapley Additive Explanations), these models offer valuable insights into the marginal contribution of each factor to price variability.

Despite growing interest in ML-based housing analysis, recent studies have applied ML methods to housing price modeling in diverse geographical contexts, such as Tehran [5] and regional South Australia [6]. In China, case studies are frequently conducted in major metropolitan or first-tier city settings, for example Shenzhen [7]. However, evidence remains limited on studies that simultaneously integrate spatial, environmental, and infrastructural variables at scale and apply interpretable ML tools to emerging second-tier cities in China.

Despite growing interest in ML-based housing analysis, most existing studies focus on first-tier cities or lack spatial granularity [7]. There remains a critical shortage of high-resolution, well-integrated datasets covering property-level, environmental, and infrastructural indicators in second-tier or emerging cities. These data limitations have hindered the translation of computational models into practical planning and policy tools. Nanning—strategically positioned under China's Belt and Road Initiative—offers a timely and representative context to investigate how machine learning can support evidence-based urban policy in a dynamic institutional setting.

This study contributes by assembling a comprehensive and multi-source dataset of over 2500 residential communities in Nanning and applying an interpretable ML framework to examine the spatial and environmental determinants of housing prices. It addresses key data gaps in sustainable urban policy research and enhances understanding of the localized mechanisms driving housing affordability and market volatility.

## 3 Data Description

### 3.1 Study area and data sources

This study collected and integrated multi-source datasets from Nanning City, a rapidly urbanizing metropolitan area in Guangxi Province, China, characterized by diverse environmental and infrastructural dynamics. Data sources included publicly available geographic databases, government spatial records, air quality monitoring stations, remote sensing datasets, and web-scraped real estate transaction information. We selected 2018 as the anchor year because it was the latest period with sufficiently complete and internally consistent housing-listing records, geocoded community identifiers, and multi-source built-environment variables after deduplication and quality control. Population density was taken from LandScan 2023 to maximize spatial completeness and harmonized grid quality. We therefore treat population density as a structural contextual covariate rather than a contemporaneous market shock variable.

### 3.2 Variable classification and description

To facilitate a clear understanding of the dataset structure, variables are categorized into five logical groups based on their attributes and relevance, with reference to the INDEX provided in the dataset. This classification helps highlight the key dimensions influencing housing prices and ensures a systematic presentation of information.

Table 1 reports summary statistics (count, mean, standard deviation, min, 25th/50th/75th percentiles, max) for all 27 numeric variables, alongside variable type, unit, and data source. Table 2 lists missing counts and percentages; the largest gaps are in PF (1681), FAR (1529), GR (1528), DIST_SUBWAY (962), DIST_COLLEGE (812), and AGE (784), while core variables (AHP, LONG, LAT, SCH_1KM) are complete. The dataset covers 2517 residential communities. Using the 1st–99th percentile trimmed extent, coordinates span 108.192–109.113°E and 21.586–23.214°N, an approximate bounding box of 94.5 km × 181.2 km (∼17,124 km²). See Table 1 for summary statistics and Table 2 for missingness.

**Table 1:** Variable definitions, units, sources, and descriptive statistics (n=2517).

Variables	Count	Mean	Std	Min	25%	50%	75%	Max
LONG	2517	108.36	0.36	107.69	108.31	108.34	108.36	123.40
LAT	2517	22.79	0.56	21.41	22.80	22.82	22.83	41.80
AHP	2517	9822.61	3415.68	0	7866.00	9240	11,709.00	30,544.00
AGE	1733	18.78	7.57	3.00	13.00	19.00	24.00	124.00
FAR	988	3.78	2.74	0.02	2.40	3.15	4.20	31.60
GR	989	0.33	0.08	0	0.30	0.35	0.35	0.90
PF	836	1.15	0.62	0.05	0.75	1.01	1.50	6.50
SCH_1KM	2517	32.50	21.81	0	19.00	28.00	42.00	167.00
DIST_KINDERGARTEN	2466	0.24	0.17	0.01	0.12	0.20	0.31	1.00
DIST_ES	2284	0.45	0.23	0.01	0.27	0.42	0.62	1.00
DIST_MS	2216	0.41	0.22	0	0.24	0.37	0.56	1.00
DIST_COLLEGE	1705	0.50	0.26	0	0.28	0.49	0.70	1.00
DIST_MEDICAL	2254	0.40	0.23	0	0.22	0.37	0.55	1.00
DIST_MALL	2141	0.42	0.24	0.01	0.22	0.39	0.60	1.00
DIST_BUS	2490	0.17	0.12	0	0.09	0.15	0.22	0.94
DIST_SUBWAY	1555	0.52	0.25	0.03	0.33	0.51	0.72	1.00
DIST_HOSPITAL	2406	0.98	0.70	0.01	0.42	0.70	2.00	2.00
HOSP_1KM	2406	5.74	7.82	0	0	2.00	7.00	40
NUM_OFFICE	2406	22.13	19.21	0	7.00	15.00	34.00	83.00
DIST_OFFICE	2406	0.39	0.43	0	0.14	0.26	0.47	2.00
AQI	2406	39.79	1.02	36.69	39.42	39.85	40.33	43.18
WATER_1KM	2406	187,853.12	276,207.82	0	0	35,497.53	294,779.52	1384,403.81
VEG_1KM	2406	59,630.31	133,794.75	0	0	2399.83	53,471.28	1894,868.35
GRASS_1KM	2406	17,151.76	42,508.96	0	0	699.95	10,174.29	503,964.99
POP_DENSITY	2406	22,401.28	23,168.95	0.41	3518.97	14,131.22	36,314.20	111,027.99
DIST_DISTRICT	2406	3.01	2.36	0.04	1.74	2.67	3.57	42.72
DIST_CITY	2406	8.45	12.08	0.11	3.50	6.24	8.93	111.48

**Table 2:** Missing values by variable (count, %).

Variables	Missing	Missing_percent(%)
CN	0	0
LONG	0	0
LAT	0	0
AHP	0	0
AGE	784	31.15
FAR	1529	60.75
GR	1528	60.71
PF	1681	66.79
SCH_1KM	0	0.00
DIST_KINDERGARTEN	51	2.03
DIST_ES	233	9.26
DIST_MS	301	11.96
DIST_COLLEGE	812	32.26
DIST_MEDICAL	263	10.45
DIST_MALL	376	14.94
DIST_BUS	27	1.07
DIST_SUBWAY	962	38.22
DIST_HOSPITAL	111	4.41
HOSP_1KM	111	4.41
NUM_OFFICE	111	4.41
DIST_OFFICE	111	4.41
AQI	111	4.41
WATER_1KM	111	4.41
VEG_1KM	111	4.41
GRASS_1KM	111	4.41
POP_DENSITY	111	4.41
DIST_DISTRICT	111	4.41
DIST_CITY	111	4.41

#### 3.2.1 Basic community identification information

This group includes variables that serve as fundamental identifiers for each residential community, laying the groundwork for data management and spatial analysis.

**Community Name (CN): A** categorical variable representing the unique name of each residential community.

**Longitude (LONG): A** continuous variable indicating the geographic longitude coordinate of the community (in degrees).

**Latitude (LAT): A** continuous variable indicating the geographic latitude coordinate of the community (in degrees).

#### 3.2.2 Core price and building characteristics

This group focuses on the target variable (housing price) and physical attributes of the residential properties, directly related to the intrinsic value of the housing.

**Average Housing Price (AHP): A** continuous variable representing the mean price per square meter of residential properties in the community (in yuan).

**Age of the House (AGE): A** continuous variable measuring the number of years since the construction of the residential property.

**Floor Area Ratio (FAR): A** continuous variable calculated as the ratio of total floor area of all buildings to the total land area in the community.

**Green Rate (GR): A** continuous variable representing the proportion of the community's area covered by greenery (ranging from 0 to 1).

**Property Fees (PF): A** continuous variable indicating monthly or yearly maintenance fees (in yuan per square meter) charged for communal services.

**Fig. 1:**
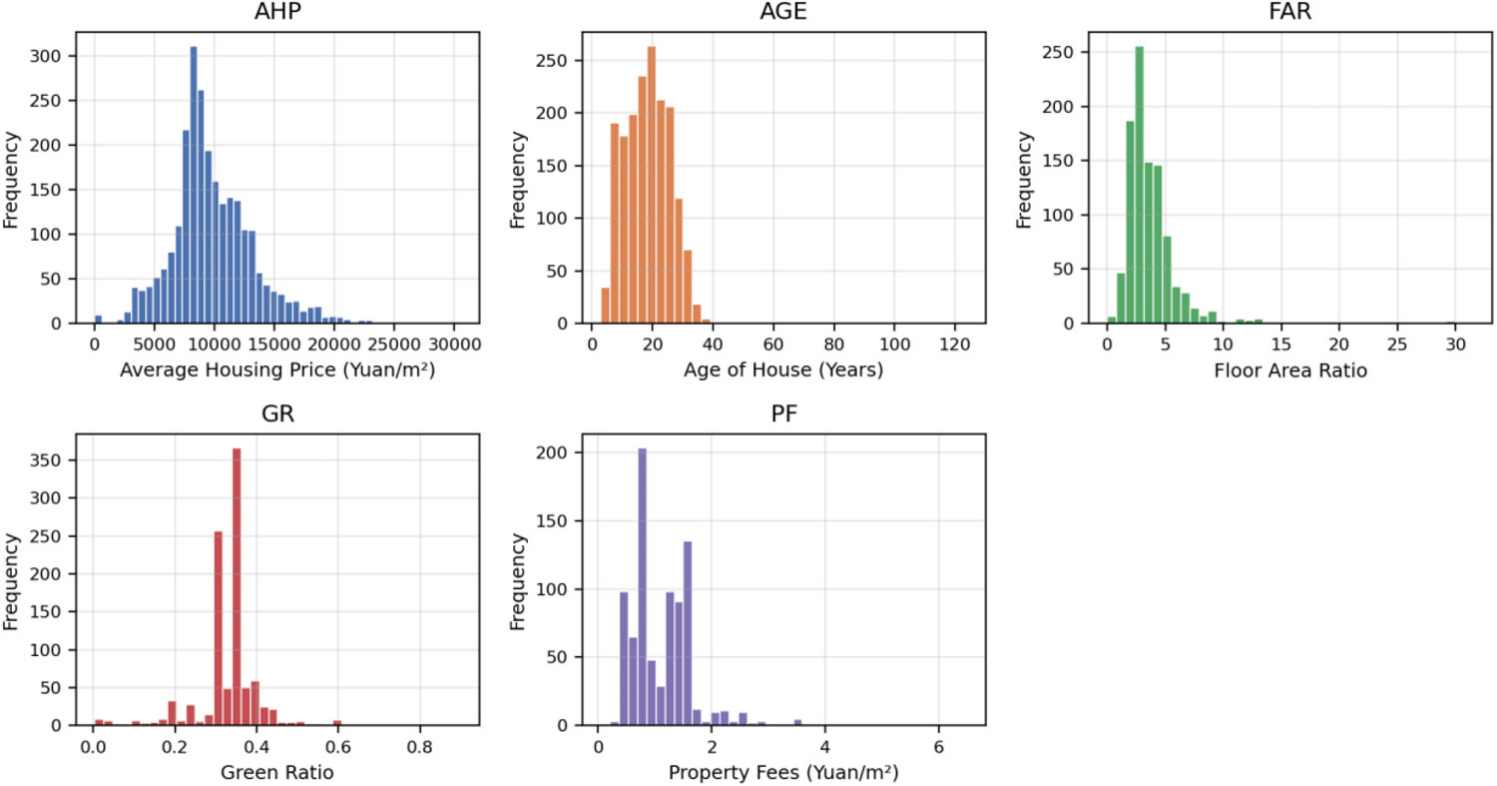
Housing price and building characteristics.

Fig. 1 presents the distribution characteristics of core price and building variables across the 2517 residential communities in Nanning. The average housing price exhibits considerable variation, with a mean of 9823 yuan/m² and a standard deviation of 3416 yuan/m². The distribution shows right skewness, with 50% of properties priced below 9240 yuan/m² and the upper quartile reaching 11,709 yuan/m². Housing age is roughly centered around 19 years, with most buildings constructed within the past two decades. The floor area ratio averages 3.78 (SD 2.74), indicating moderate development density with substantial variation. Green space coverage is relatively consistent (mean 0.33, SD 0.08). Property fees average 1.15 yuan/m² monthly, suggesting standardized maintenance costs across community types. All univariate plots use the same histogram style (including AGE and count variables).

#### 3.2.3 Educational and medical support facilities

This group encompasses variables related to public services such as education and healthcare, which are critical for family-oriented housing choices and quality of life.

**Number of Schools in 1** **km (SCH_1KM): A** continuous variable counting the number of educational institutions (kindergartens, elementary, middle schools) within a 1-km radius.

**Distance to the Nearest Kindergarten (DIST_KINDERGARTEN): A** continuous variable measuring the straight-line distance (in kilometers) to the nearest kindergarten.

**Distance to the Nearest Elementary School (DIST_ES):** A continuous variable indicating the straight-line distance (in kilometers) to the nearest elementary school.

**Distance to the Nearest Middle School (DIST_MS):** A continuous variable representing the straight-line distance (in kilometers) to the nearest middle school.

**Distance to the Nearest College (DIST_COLLEGE): A** continuous variable measuring the straight-line distance (in kilometers) to the nearest college or higher education institution.

**Distance to the Nearest Medical Institution (DIST_MEDICAL):** A continuous variable indicating the straight-line distance (in kilometers) to the nearest medical facility (clinics or hospitals).

**Distance to the Nearest General Hospital (DIST_HOSPITAL):** A continuous variable representing the straight-line distance (in kilometers) to the nearest general hospital.

**Number of General Hospitals in 1** **km (HOSP_1KM): A** continuous variable counting the number of general hospitals within a 1-km radius.

**Fig. 2:**
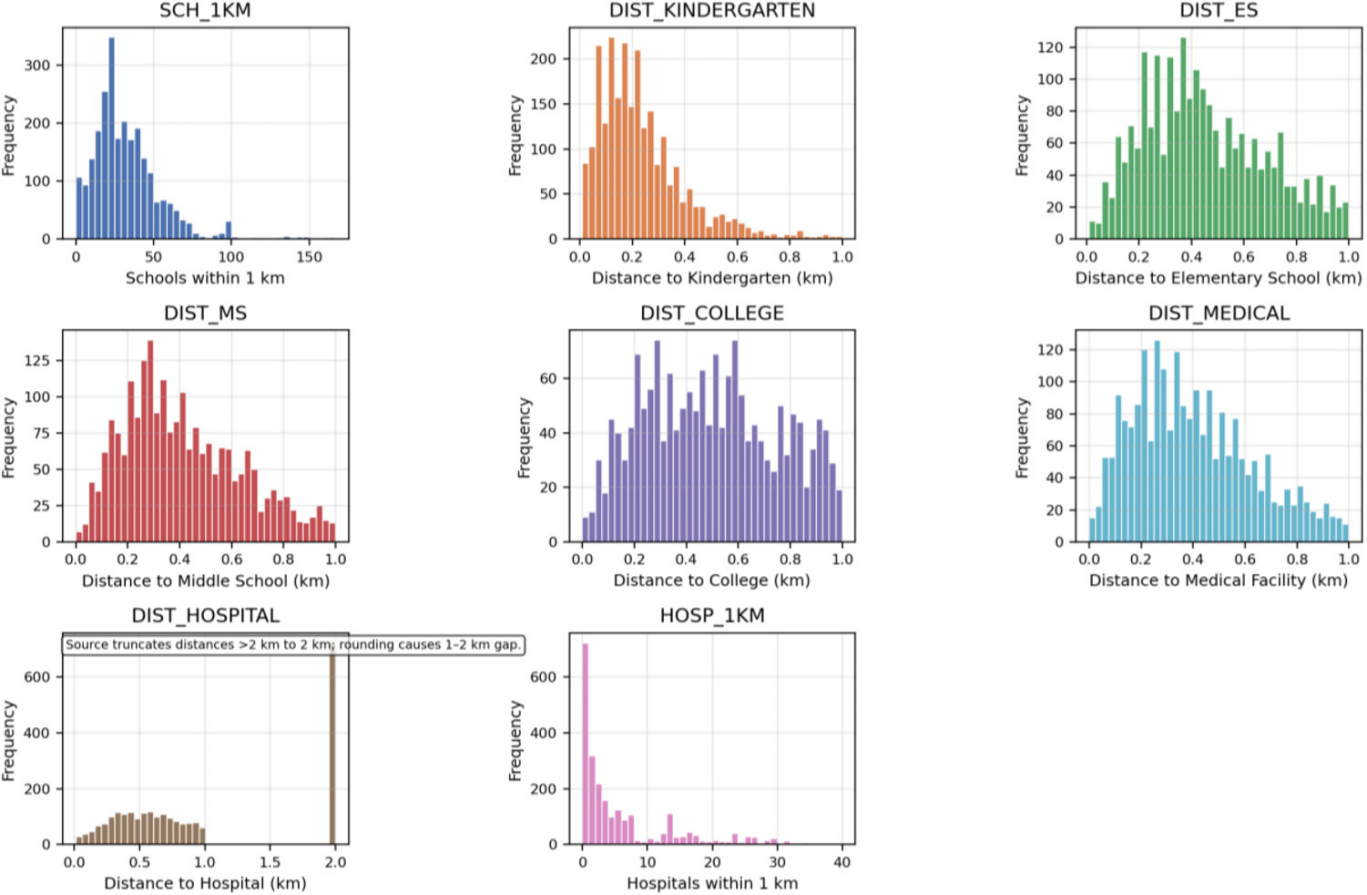
Educational and medical support facilities.

The educational and medical infrastructure variables demonstrate significant spatial heterogeneity across Nanning's residential areas (Fig. 2). School accessibility varies considerably, with communities averaging 33 educational institutions within a 1‑kilometer radius (range 0–167). Distance-based measures show generally good access—kindergartens are typically within 240 m and elementary schools within 450 m—though right-skewed distributions indicate peripheral areas with weaker access. DIST_HOSPITAL shows a spike at 2 km because the source data truncate distances beyond 2 km and some values were rounded, leaving a gap between 1 and 2 km. Medical facility access is similar, with healthcare institutions averaging 400 m from communities and hospital density within 1‑kilometer buffers highly variable (mean 5.74, SD 7.82), reflecting clustered specialized services. These patterns underscore the importance of educational and healthcare proximity in residential location choices.

#### 3.2.4 Commercial and transportation facilities

This group includes variables related to daily life convenience, such as commercial services and public transportation, which are key to residents' quality of life.

**Distance to the Nearest Shopping Mall (DIST_MALL): A** continuous variable measuring the straight-line distance (in kilometers) to the nearest shopping mall.

**Distance to the Nearest Bus Stop (DIST_BUS):** A continuous variable indicating the straight-line distance (in kilometers) to the nearest public bus stop.

**Distance to the Nearest Subway (DIST_SUBWAY):** A continuous variable representing the straight-line distance (in kilometers) to the nearest subway station.

**Fig. 3:**
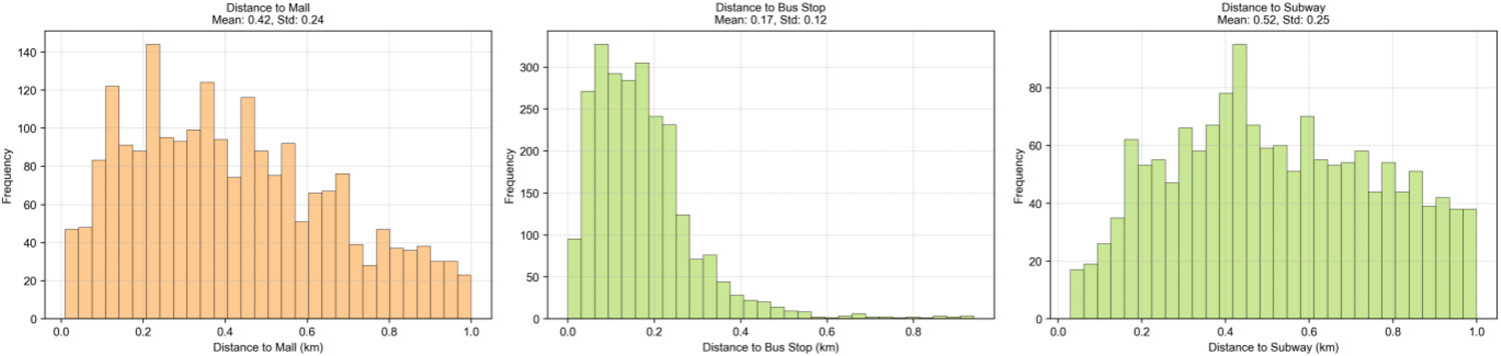
Commercial and transportation facilities.

Commercial and transportation accessibility exhibits distinct distribution patterns reflecting Nanning's urban development structure (Fig. 3). Shopping mall accessibility demonstrates moderate variation, with residential areas averaging 420 m from the nearest mall and a standard deviation of 240 m The distribution suggests relatively even commercial development across the city, with 75% of communities located within 600 m of shopping facilities. Public transportation accessibility shows excellent coverage, particularly for bus services, with communities averaging only 170 m from the nearest bus stop. This exceptionally high accessibility reflects Nanning's comprehensive public bus network. Subway accessibility presents greater variation, averaging 520 m with a wider distribution range, indicating that metro coverage remains more concentrated in central urban areas. The interquartile range for subway distance (330–720 m) suggests that while central communities enjoy excellent metro access, peripheral developments may rely more heavily on bus transportation.

#### 3.2.5 Location, environment, and population characteristics

This group covers variables reflecting macro-level location advantages, environmental quality, and demographic features, which influence long-term housing market performance.

**Number of Office Buildings (NUM_OFFICE): A** continuous variable counting the total number of office buildings in the vicinity of the community.

**Distance to the Nearest Office Building (DIST_OFFICE):** A continuous variable measuring the straight-line distance (in kilometers) to the nearest office building.

**Air Quality Index (AQI): A** continuous variable representing the average air quality index, with lower values indicating better air quality.

**Water Area in 1** **km (WATER_1KM): A** continuous variable measuring the total area (in square meters) of water bodies (lakes, ponds) within a 1-km radius.

**Vegetation Area in 1** **km (VEG_1KM): A** continuous variable indicating the total area (in square meters) covered by vegetation within a 1-km radius.

**Grassland in 1** **km (GRASS_1KM): A** continuous variable representing the total area (in square meters) of grassland within a 1-km radius.

**Surrounding Population Density (POP_DENSITY): A** continuous variable measuring the average population density (people per square kilometer) in the vicinity.

**Distance to District Center (DIST_DISTRICT):** A continuous variable indicating the straight-line distance (in kilometers) to the administrative district center.

**Distance to City Center (DIST_CITY):** A continuous variable representing the straight-line distance (in kilometers) to the city center.

**Fig. 4:**
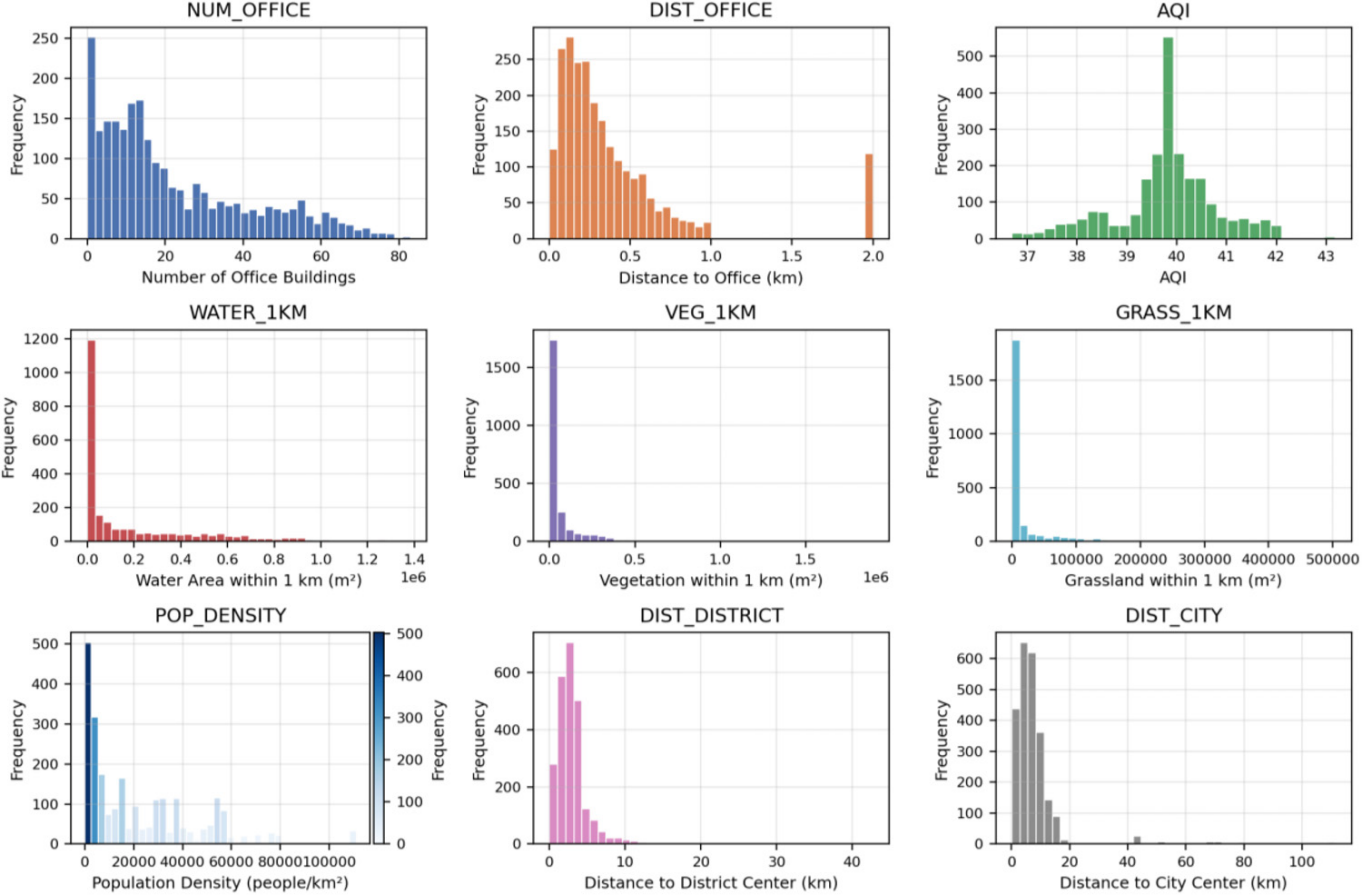
Location, environment, and population characteristics.

The location, environmental, and population characteristics reveal the complex spatial organization of Nanning's urban landscape (Fig. 4). Office building density shows substantial variation across residential areas, averaging 22 buildings per community with a standard deviation of 19, indicating concentrated business districts alongside residential zones. Environmental quality variables demonstrate considerable spatial heterogeneity, with air quality showing relatively low variation (mean AQI of 39.8, standard deviation of 1.0), suggesting consistent environmental conditions across the study area. Water and vegetation coverage exhibit highly skewed distributions, with many communities having minimal green or water features (median values near zero), while others benefit from substantial environmental amenities. Population density displays remarkable variation, ranging from 0.4 to 111,028 people per square kilometer, with a mean of 22,401 and median of 14,131, highlighting the contrast between high-density urban cores and lower-density suburban areas; the population-density histogram includes a color legend indicating frequency. Distance to administrative and commercial centers shows expected patterns, with communities averaging 3.0 km from district centers and 8.5 km from the city center, though substantial variation exists reflecting the city's polycentric development pattern.

## 4 Experimental Design, Materials and Methods

### 4.1 Data collection and preprocessing

Residential property data, including detailed attributes (e.g., property age, floor area, property type), were obtained through web scraping from popular real estate platforms such as Lianjia, Fang.com, and Anjuke,which together cover a substantial portion of online second-hand housing advertisements in major cities. Even though this list is not strictly exhaustive, as other local or emerging platforms may also publish listings; however, these three platforms were selected because they are consistently available in Nanning, provide relatively standardized fields (e.g., price, floor area, floor level, building age, community name), and are commonly adopted in empirical housing-market studies. To reduce potential platform-specific biases, we combined records from multiple sources whenever overlapping listings were available. Besides, duplicate records were removed using a rule-based multi-key matching approach. Candidate duplicates were identified when (i) the community name and administrative district were identical after normalization, and (ii) floor area and price variables were highly similar within predefined tolerances. For candidate groups, we compared geocoded coordinates to ensure community-level consistency. When duplicates were confirmed, only one record was retained to avoid repeated counting of the same property across platforms or reposting periods.

Ethical guidelines for web scraping were strictly observed, including careful anonymization and aggregation of personal data to ensure privacy protection. Each property's name and associated administrative region were extracted and utilized for subsequent geocoding.

We cross-checked key community-level attributes (community name, district assignment, and geocoded coordinates) against authoritative sources (government administrative divisions and map-service geocoding). The validation indicated high agreement: most communities were matched successfully, and geocoded coordinates showed small spatial discrepancies at the community scale. Listings that could not be matched reliably due to ambiguous names or inconsistent district/location information were removed to ensure data quality. Perfect year-level synchronization is not feasible across all sources in multi-source urban datasets. In this study, housing outcomes are anchored to 2018, while population density uses the 2023 LandScan layer. Because population distribution at community scale changes more gradually than listing prices, this variable is used to characterize long-run spatial context. We acknowledge that this mismatch may introduce measurement error and therefore interpret estimated effects conservatively.

**Fig. 5:**
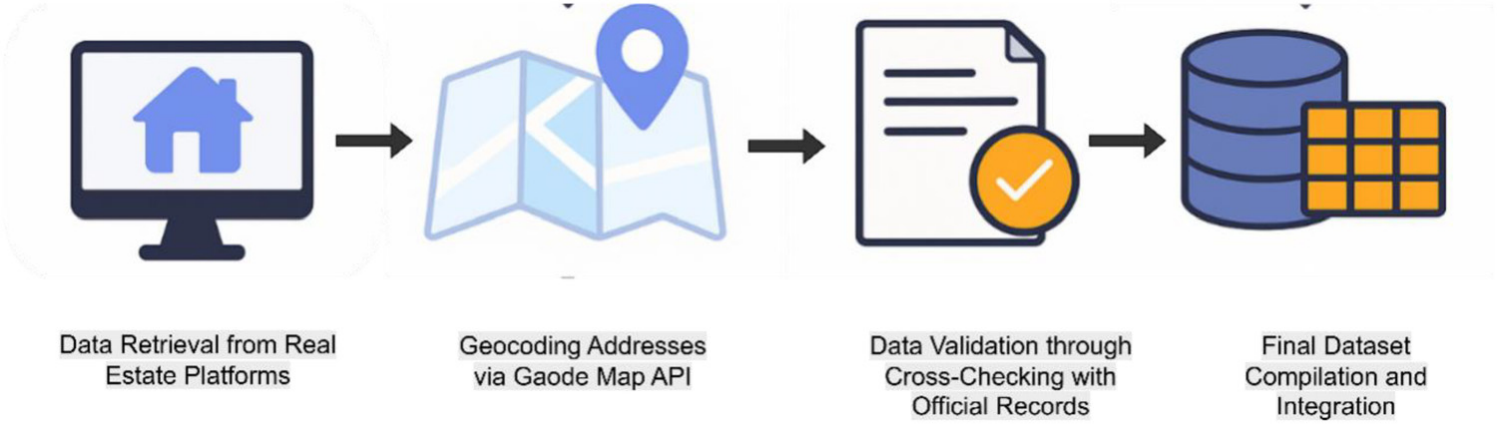
Workflow of geocoding and community-level matching.

As shown in Fig. 5, housing listings were scraped from online platforms, cleaned and deduplicated, then geocoded to coordinates using map-service geocoding. Listings were aggregated into residential communities, and community-level features were constructed by linking multi-source urban facility POIs and environmental data to each community. Finally, all validated data were compiled and integrated into a final structured dataset for subsequent analysis.

### 4.2 Spatial feature construction

**Fig. 6:**
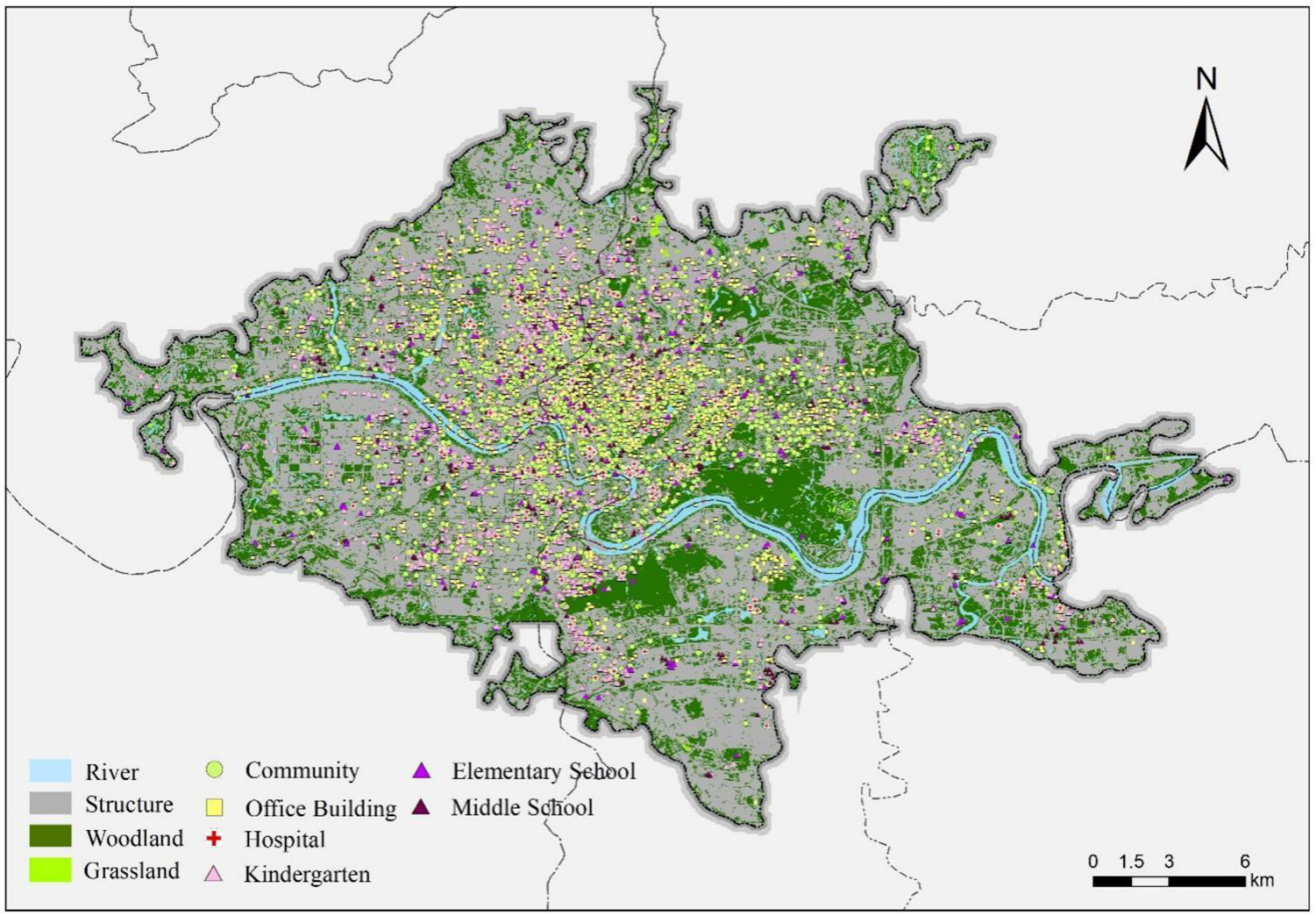
Map of study area showing property locations and key urban facilities.

**Fig. 7:**
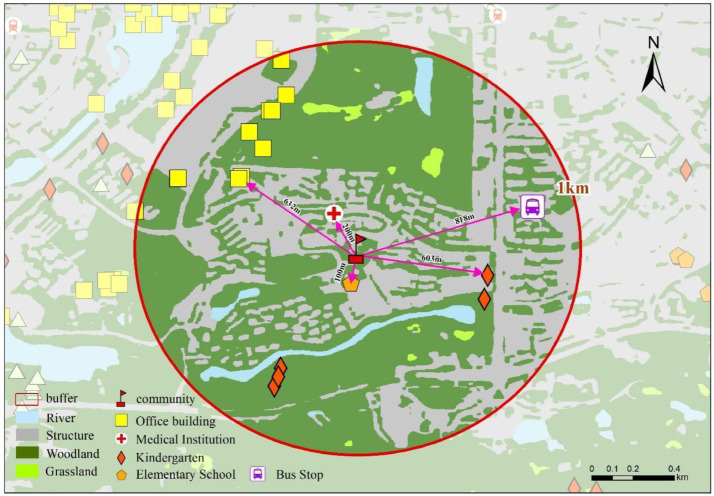
Distance calculations to urban amenities.

**Fig. 8:**
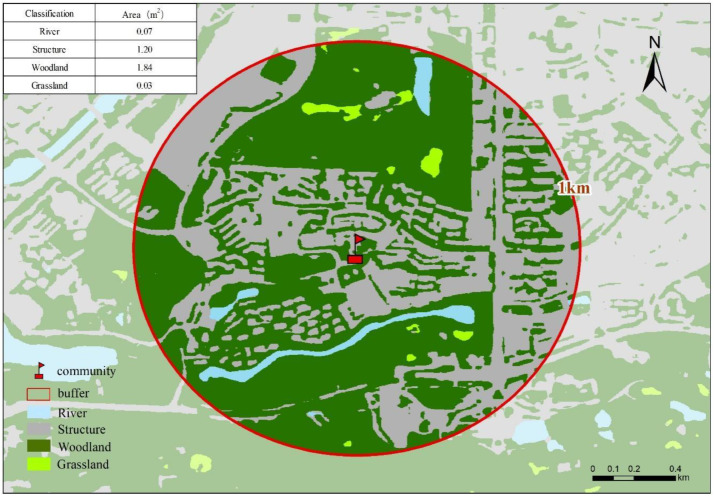
Illustration of spatial buffer, proximity analysis, and land-use data extraction in GIS.

The geographic locations of urban facilities (including hospitals, schools, shopping malls, subway stations, and major office buildings) were obtained from publicly accessible point-of-interest (POI) sources and verified through mapping services (Fig. 6). Specifically, facility coordinates and categories were retrieved using a map-service POI interface (e.g., Gaode/AMap POI search and geocoding functions) based on standardized keyword queries and category filters. To improve robustness, we performed manual spot-checking for a subset of facilities with uncertain naming or category assignment (e.g., facilities with multiple branches or similarly named institutions). The final POI database was then used to compute accessibility indicators such as nearest-distance and facility counts within specified buffers. The number of facilities within a 1-km radius was counted and recorded (Fig. 7, Fig. 8).

Environmental variables included land use types, derived from authoritative governmental land use documents at fine spatial resolutions. GIS analysis involved creating a 1-km buffer around each property to calculate the proportions of different land use categories, such as green spaces, water bodies, and urban infrastructure.

**Fig. 9:**
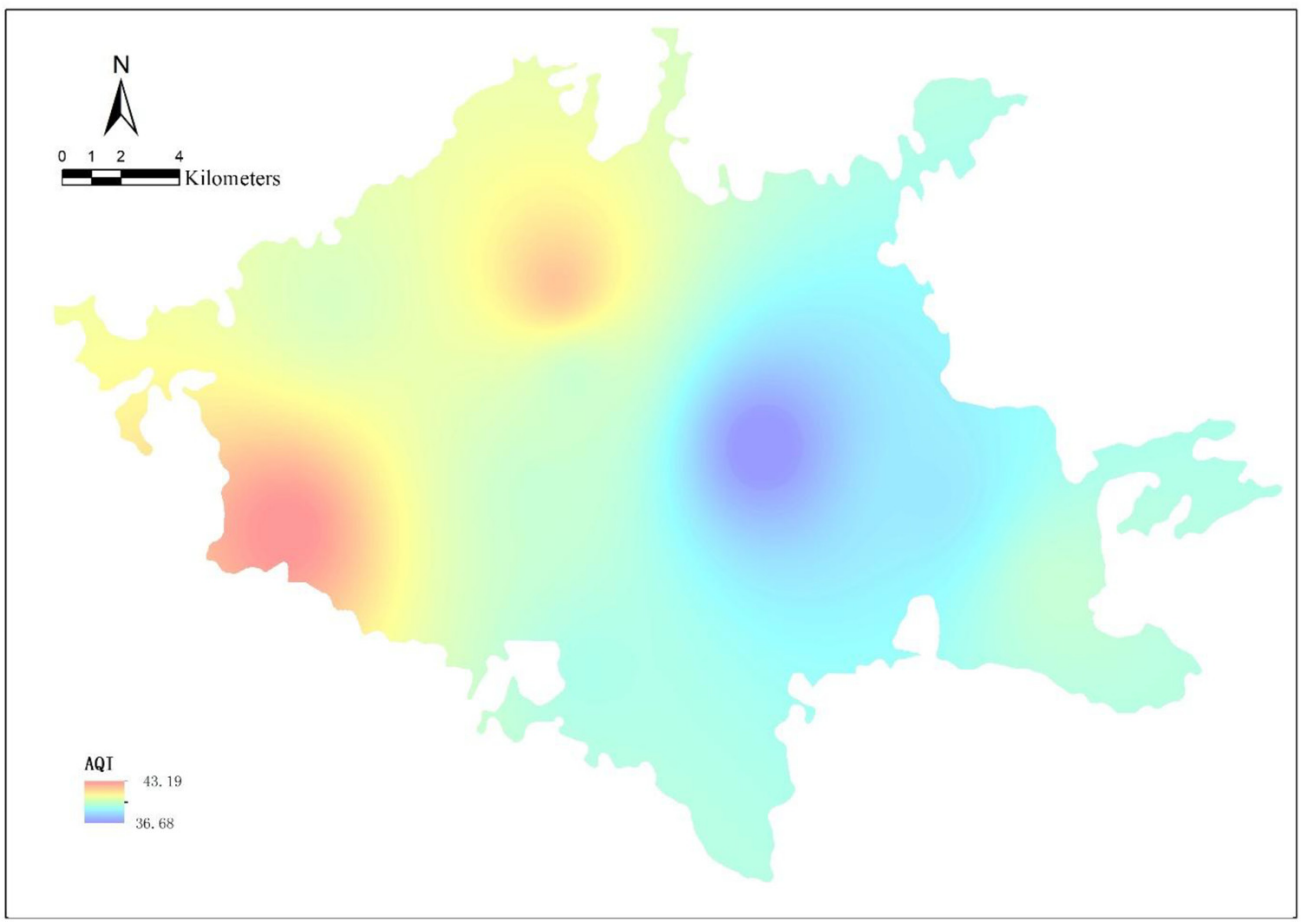
Spatial interpolation (IDW) of AQI values with monitoring station locations.

Air quality was represented using the Air Quality Index (AQI) derived from official environmental monitoring stations across Nanning. Inverse Distance Weighting (IDW) interpolation, a validated and widely used spatial interpolation technique, was applied to estimate AQI values at property locations (Fig. 9).

**Fig. 10:**
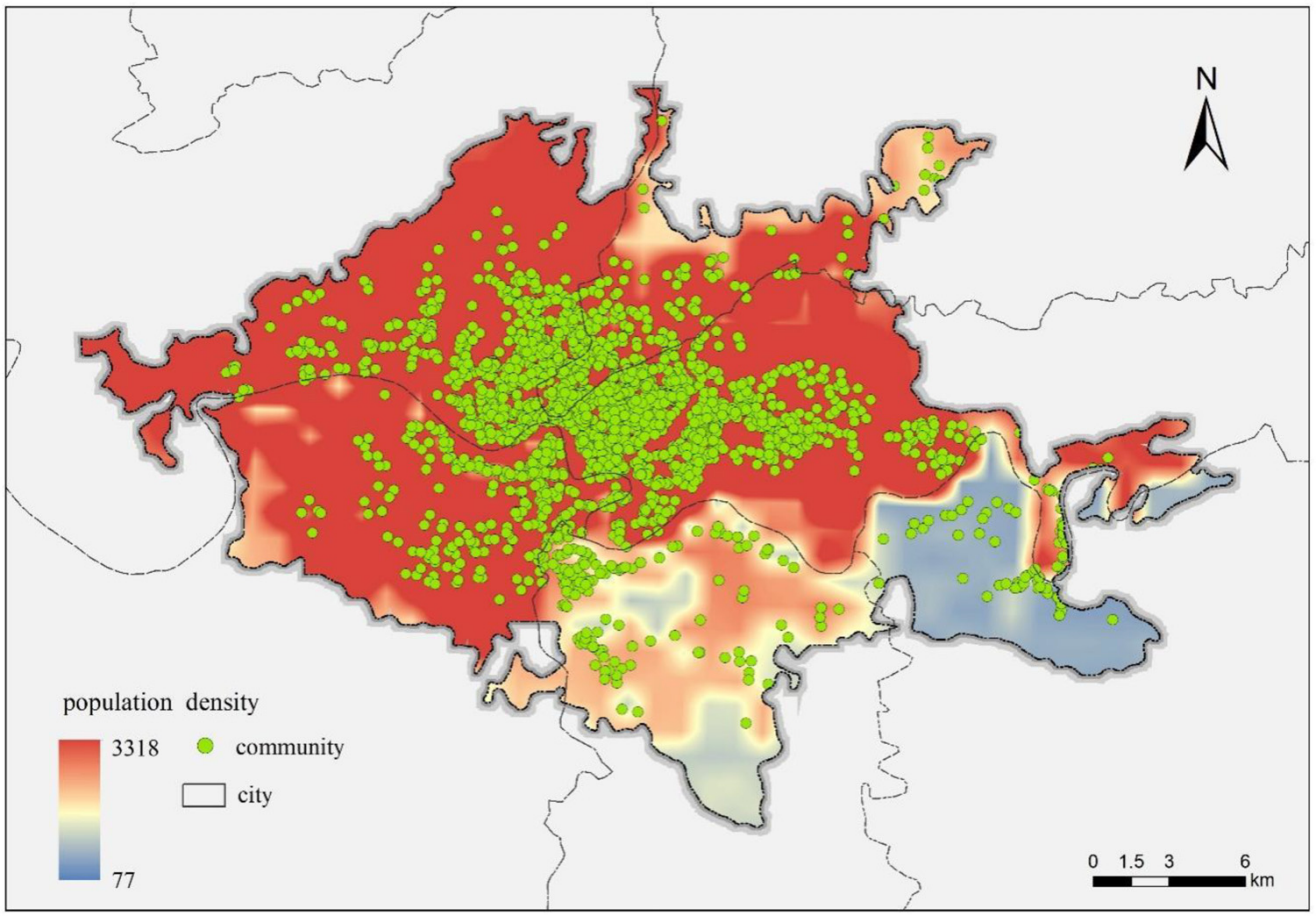
GIS-based population density.

Population density data were extracted from the LandScan dataset, developed by Oak Ridge National Laboratory (ORNL). The LandScan dataset provides high-resolution (1 km × 1 km) annual population estimates, extensively validated for neighborhood-scale applications. Population density values at each property location were extracted using spatial overlay operations in GIS (Fig. 10).

### 4.3 Variable selection and justification

The feature system was designed following established housing-price modeling practices, particularly the hedonic pricing framework [8,9], which decomposes property value into structural characteristics, neighborhood attributes, and accessibility-related factors. In line with this framework, this study organized explanatory variables into categories below:

**Property characteristics**: Variables such as property age, building type, and floor area were chosen based on established real estate valuation theories that directly link structural attributes to market values.

**Accessibility to public amenities**: Distance to hospitals, schools, malls, subway stations, and other facilities were included given their recognized importance in shaping housing values due to convenience and urban livability.

**Landscape and environmental features**: Variables representing proximity and proportion of green space, water bodies, and air quality were selected for their demonstrated relationship to residential attractiveness, environmental quality, and public health.

**Traffic and location factors**: Proximity to city centers, major transportation hubs, and road networks were chosen based on extensive literature documenting their critical role in urban property valuation.

Variables excluded, such as school quality ratings, crime rates, or road density, were acknowledged as limitations due to data availability constraints.

### 4.4 Machine learning modeling

This study trained three representative predictive models to estimate housing prices from the constructed features: Random Forest (RF), XGBoost, and Linear Regression (LR). These models were selected to provide a balanced comparison between (i) an ensemble bagging method (RF) with strong nonlinear fitting ability, (ii) a gradient-boosting method (XGBoost) widely used in tabular prediction tasks, and (iii) a classical linear baseline (LR) for interpretability and reference.

Missing data were handled using a predefined rule before model fitting. Variables with missingness greater than 60% were excluded from the primary feature set; therefore, FAR (60.75%) and PF (66.79%) were not included in the main RF/XGBoost models. For retained predictors with partial missingness, imputation was performed using training-set statistics and then applied to the test set to avoid data leakage. We additionally conducted a sensitivity analysis that reintroduced FAR and PF under imputation-only settings, and the model ranking and main substantive conclusions were unchanged.

Model performance was evaluated on the held-out test set using Mean Squared Error (MSE) and the coefficient of determination (R²). Among the evaluated models, the Random Forest achieved the best overall predictive performance and was therefore used for subsequent interpretation analyzes.

As shown in Table 3, the Random Forest model achieved the highest predictive accuracy with an R² of 0.7418, an MSE of 0.2184. This represents a significant improvement over the baseline Linear Regression model (R² = 0.6925) and a competitive edge over XGBoost (R² = 0.6870), confirming the Random Forest model's suitability for capturing the non-linear dynamics of the Nanning housing market.

**Table 3:** Performance comparison of machine learning models for housing price prediction [1].

Model	Mean Squared Error (MSE)	R-squared (R^2^)
Random Forest (Proposed)	**0.2184**	**0.7418**
XGBoost	0.2927	0.6870
Linear Regression	0.2889	0.6925

The optimal hyperparameters determined were n_estimators=192, max_depth=10, and min_samples_split=20, providing a balance between model complexity and generalization.

### 4.5 SHAP interpretability analysis

To explain feature contributions, we applied **SHAP (SHapley Additive exPlanations)** to quantify the marginal impact of each variable on model predictions at both global and local levels. The SHAP summary plots and dependence patterns provide transparent insights into the main determinants of price variation and their nonlinear effects in the study area.

**Summary of ML workflow.** The overall pipeline consists of (1) web-scraping and cleaning housing listings, (2) geocoding and linking each listing to residential communities, (3) constructing multi-source explanatory features (structure, neighborhood, accessibility, environment), (4) train–test splitting and cross-validation-based hyperparameter tuning, (5) model evaluation using MSE and R², and (6) SHAP-based interpretation of the best-performing model.

## 5 Limitations

This dataset, while comprehensive, has several inherent limitations that may affect the interpretation and generalization of research results:

### 5.1 Variable measurement and data accuracy

Distance variables are measured as straight-line distances, which may not accurately reflect actual travel routes affected by road networks, traffic patterns, and topographical barriers. Some variables contain missing values, particularly for distance to colleges, which may reduce statistical power and introduce potential sample bias. The dataset focuses on objectively quantifiable indicators while excluding subjective factors such as residents' satisfaction with community environments and perceived quality of surrounding facilities.

### 5.2 Data timeliness and dynamic changes

This cross-sectional dataset reflects housing market conditions at a specific point in time, unable to capture dynamic changes such as new infrastructure development, urban planning adjustments, or macroeconomic fluctuations. Policy impacts may not be timely reflected due to data collection lag, limiting the dataset's ability to predict policy-induced market changes. This dataset is a cross-sectional snapshot centered on 2018 and cannot capture year-to-year market dynamics. In addition, population density is sourced from 2023 due to data availability and standardized coverage constraints. Although this variable is used as a structural contextual control, cross-year mismatch may attenuate estimated associations and limits strict causal interpretation. Future releases will prioritize year-matched population surfaces or panel harmonization.

### 5.3 Sample representativeness and extrapolation

The dataset is geographically limited to Nanning, with findings that may not be directly applicable to other cities with different economic, demographic, or cultural contexts. Community type coverage may be incomplete, potentially underrepresenting certain housing segments such as affordable housing or luxury villa communities, which could limit comprehensive understanding of the entire housing market spectrum.

## Ethics Statement

The authors declare that they have read and followed the ethical requirements for publication in Data in Brief. This article does not involve studies with human participants, animals, or data collected from social media platforms.

## Credit Author Statement

**Wenbo Li**: Methodology, Original draft preparation. **Kaichuang Wu**: Data curation, Writing. **Xiaolu Zhang**: Visualization, Software. **Xiaoling Gu**: Software, Investigation. **Jun Wang**: Validation. **Ming Jiang**: Writing- Reviewing and Editing. **Feng Han**: Supervision, Conception.

## Data Availability

Mendeley DataDataset on Price and basic information of residential units in Nanning (Original data).
